# Urban rhythms in a small home: COVID-19 as a mechanism of exception

**DOI:** 10.1177/00420980211018136

**Published:** 2021-06-27

**Authors:** Jenny Preece, Kim McKee, David Robinson, John Flint

**Affiliations:** University of Sheffield, UK; University of Stirling, UK; University of Sheffield, UK; University of Sheffield, UK

**Keywords:** housing, inequality, neighbourhood, small homes, urban rhythms

## Abstract

The amount of living space we have access to is one manifestation of the unequal distribution of housing resources within societies. The COVID-19 pandemic has required most households to spend more time at home, unmasking inequalities and reigniting longstanding debates about the functionality and experience of smaller homes. Drawing on interviews across three UK cities, this article attends to the changing household routines of individuals living in different types of small home, exploring daily life before and during ‘lockdown’. Using the concept of urban rhythms, the data show that the lockdown has intensified existing pressures of living in a smaller home – lack of space for different functions and household members – whilst constraining coping strategies, like spending time outside the home. Lockdown restrictions governing mobility and contact acted as a mechanism of exception, disrupting habitual patterns of life and sociability, and forcing people to spend more time in smaller homes that struggled to accommodate different functions, affecting home atmospheres. For some, the loss of normal strategies was so significant that they sought to challenge the new rules governing daily life to protect their wellbeing.

## 摘要

我们所能获得的居住空间的大小是社会内部住房资源分配不平等的一种表现。新冠肺炎大流行迫使大多数家庭花更多时间呆在家里，这凸显了住房不平等问题，并重新点燃了关于狭小住宅的功能和体验方面的长期争论。基于在英国三个城市开展的访谈，本文关注生活在不同类型的狭小住宅里的个人不断变化的常规，探讨了“封锁”之前和封锁期间的日常生活。利用城市节奏的概念，我们的数据显示封锁加剧了居住在狭小住宅（缺乏足够的空间满足不同功能和不同家庭成员的需要）中的个人的既有压力，同时限制了应对策略（如在户外度过时间）的应用。限制行动和联系的封锁措施形成了一种例外机制，扰乱了习惯性的生活和社交模式，迫使人们在各类功能捉襟见肘的狭小住宅内度过更多时间，家庭氛围也因此受影响。对一些人来说，无法执行通常策略所带来的损失是如此巨大，以至于他们试图挑战新的日常生活管理规则以保护自己的福祉。

## Introduction

Space in the home is an important part of health and wellbeing (Carmona et al., 2010). However, it has been shrinking since the 1970s (LABC Warranty, 2019) and England has among the smallest homes in Western Europe, with over a third of households short of space (Morgan and Cruickshank, 2014). This has fuelled debates about small homes in the UK, and internationally, particularly in relation to new homes (Lau and Wei, 2018; Tervo and Hirvonen, 2020). Critics note that smaller homes can be inflexible (CABE, 2009; Gallent et al., 2010), engender dissatisfaction even when not fully occupied (Morgan and Cruickshank, 2014) and can negatively impact on wellbeing (Brown et al., 2020). This article explores the negotiation and adaptive strategies of life in a small home, demonstrating that the COVID-19 pandemic intensified already existing challenges by requiring individuals to stay at home and reduce physical contact between households. Data are drawn from 27 in-depth interviews with individuals in three UK cities – London, Sheffield and Edinburgh – living in different types of smaller home.

During the initial UK ‘lockdown’, from 23 March 2020, individuals were only permitted to leave their home to shop for essential items (e.g. food), for exercise once a day, for medical needs or to assist a vulnerable household. Non-essential shops and businesses were closed and schools were shut to most children. Individuals were required to work from home, with the exception of workers critical to the COVID-19 response, such as those working in healthcare, or food production and distribution. Restrictions on the number of daily exercise periods were lifted from 13 May, and at the time of the interviews in June 2020 the stay-at-home directive was replaced with permission to gather outside in groups of up to six individuals in England, or two households comprising eight individuals in Scotland (maintaining a distance of 2 m). From late June, retail shops in England and Scotland reopened.

The distribution of space in the home is a manifestation of wider housing inequalities (Blackall, 2020), and COVID-19 has intensified existing debates about small-home living. This study responds to calls from Wiles (2020) and Harris and Nowicki (2020) to consider how the shrinking of home spaces impacts people’s wellbeing, and the implications of the blurring of home–work–care spaces. These are particularly important issues in the context of a global pandemic in which ‘we do not know how … negative psychological effects … might be mediated by housing conditions, including the amount of living space or the presence/absence of gardens and balconies’ (Gurney, 2020: 9). National guidance pointed to inequalities in experiences of the pandemic, noting that staying at home ‘can be particularly challenging if you do not have much space or access to a garden’ (Public Health England, 2020), whilst research into overcrowding has shown that fitting different activities into a limited space may have adverse impacts (Carmona et al., 2010). Early surveys during lockdown linked lack of space in the home to health problems, depression and stress (National Housing Federation, 2020), with disproportionate impacts on mental health among young people, those living alone, with lower incomes, with a diagnosed mental illness, those living with children and those living in urban areas (Fancourt et al., 2020).

There is an established body of research into experiences of private rented and shared housing, which is often smaller per person (McKee et al., 2017; Soaita and McKee, 2019). Our research makes a number of contributions. First, it extends the form and type of small home to recognise a broader spectrum of small-home living, offering the potential to identify commonalities of experience on the basis of space. Existing research into small homes has focused on niche forms, such as tiny houses (Boeckermann et al., 2019; Carras, 2019) or shed housing (Lombard, 2019), which are characterised by greater informality, constraint or alternative lifestyles (Harris and Nowicki, 2020). Second, we focus on lived experience and everyday life, considering how the trade-offs of small-home living – such as size versus location (Lau and Wei, 2018) – continue to be negotiated through the ‘micro-politics’ of dwelling practices (Easthope et al., 2020). The in-depth understanding of how individuals use space in the home, generated by this research, can inform planning for future housing needs (Drury, 2008), particularly in relation to post-COVID recoveries (Judge and Rahman, 2020), which may include shifting priorities around home-working and outdoor space.

Third, conceptually we bring together the concepts of exception, rhythms and atmospheres in a novel framework. These concepts are unified by the focus on dynamic, relational processes that unfold between people and objects in specific sites. Using the concept of urban rhythms to analyse residents’ experiences, the article foregrounds the ways in which habitual patterns and routines of life have been reshaped through the ‘mechanism of exception’ (Stavrides, 2013) of lockdown under COVID-19. In doing so, the research makes a conceptual contribution by demonstrating the inter-relationship between rhythms, the spatiality of small homes and the atmospheres generated through everyday practices. The patterning of daily life was already significantly influenced by the space of the home, which the ‘exception’ of lockdown has intensified.

The article first sets out this conceptual framing, before describing the approach to undertaking qualitative interviews during lockdown. The findings show that before lockdown, everyday life in a small home was characterised by a series of particular spatial and relational negotiations. Leaving the home was a crucial coping mechanism, as well as enabling engagement with the eventfulness of city living. Lockdown exacerbated the challenges of living in a small home, whilst removing some of the adaptive mechanisms used by residents. This had practical and affective impacts, as residents shared space with others and required this space to deliver a greater number of functions, particularly in relation to work. For some, the negative impact of lockdown resulted in attempts to counter the set patterns brought about by restrictions on sociability and movement.

## Urban rhythms and daily life

Lefebvre (2004: 15) conceptualises rhythms as a way to understand the unfolding of everyday activities: ‘everywhere there is interaction between a place, a time, and an expenditure of energy, there is a rhythm’. Increasingly standardised, linear rhythms promoted greater synchronicity with the routines of industrial capitalism (Kern, 2015), including the patterning of each day and the distinction between weekends and weekdays (Aquist, 2004). Work therefore became the time of everydayness, dominating other aspects of the everyday (Lager et al., 2016). The dominance and synchronicity of these rhythms have waned with the decline of industrial production, replaced by greater diversity in working hours, sectors and location of activities (Mulíček et al., 2016); the pandemic has seen a return to the home as a site of paid labour for growing numbers of workers.

Cities are comprised of a mosaic of rhythmically diverse sub-systems that connect groups of residents and city-users (Mulíček et al., 2016). Stavrides (2013) goes further, arguing that post-industrial societies are organised by separate urban settings which are distinguished by their different rhythms. These sites are dynamic, produced through the intersection of the multiple rhythms of users and residents (Lager et al., 2016). For residents, access to the cultural life and amenities of the city is often an important part of housing choices, which may be traded off against other things, such as space. As well as work, therefore, consumption-oriented events also structure neighbourhood rhythms (Kern, 2015; Mulíček et al., 2016). This creates a particular form of street life that is available to residents who have the means to synchronise aspects of their daily life to this rhythm of ‘eventfulness’, but also the potential for disruption for those who are not able to maintain congruence with dominant rhythms (Kern, 2015; Lager et al., 2016). As Reid-Musson (2018: 885) notes:

daily work-life schedules, and the flows, frictions and stasis associated with the boundaries between leisure, work and employment, provide an empirical foothold for studying intersecting … positions … power differentials … and how risk and vulnerability are borne at the level of rhythms.

The coronavirus pandemic has unsettled these boundaries, unmasking inequalities, particularly expressed through the unequal distribution of housing.

## Lockdown, exception and home atmospheres

Restrictions on movement and social contact as a result of COVID-19 can be conceptualised as a ‘mechanism of exception’ (Stavrides, 2013), in which a form of emergency or temporary need justifies the suspension of general laws or rights, resulting in a break in established rhythms. Historically, these mechanisms of exception have usually been invoked as a means through which to protect ‘normality’ in the face of a threat, such as terrorism; thus, the temporary deviation from normal life serves to enable its reestablishment. Whilst the deviation from established routines may be experienced as an arrhythmia – a discordant and disruptive rhythm (Reid-Musson, 2018) – the shift can also in some cases transition to a permanent readjustment as new rhythms become normalised (Stavrides, 2013). However, there are also opportunities for counter-rhythms and disruption (Reid-Musson, 2018), in which individuals resist dominant rhythms of exception and insert ‘hidden rhythmicalities of survival’ (Stavrides, 2013: 42). In a time of lockdown, this may include socialising with other households, or leaving the home for ‘non-essential’ reasons.

The lockdown reshaped the normative frameworks through which daily urban rhythms have been understood (Reid-Musson, 2018), challenging durable practices and the ‘rules’ governing behaviour and social relations (Burkitt, 2004). Restrictions to movement reflected a new regulation of mobility, which redefined ‘correct’ movements through space (Cresswell, 2010). This shift is inherently spatial, requiring an understanding of the intersection of practices and place (Pink, 2012). As part of the process of coming to know the world around them, individuals seek sites that support and sustain their everyday practices (Duff, 2010); whilst this is often unconscious and habitual, contributing to a sense of ‘feeling right’ in particular places (Lager et al., 2016; Pink, 2012), the rapid changes wrought by responses to COVID-19 have forced sites such as the home to accommodate reconfigured activities and occupational practices. The transformation of daily life at the level of rhythms therefore has a potential impact on practices within the home, and relatedly the affective experience of home.

If there are rhythms everywhere, so too there are atmospheres, which form part of the backdrop to daily life, often unnoticed or habitual in nature, but affecting how we inhabit spaces. Atmospheres can be thought of as collective affects that occur ‘across human and non-human materialities andin-between subject/object distinctions’, representing a shared encounter which creates subjective states, feelings and emotions (Anderson, 2009: 78). They can be produced by various means, including through common rhythms, expressed through specific encounters between people, objects and sites (Anderson, 2016). Therefore, the rhythms that pattern everyday life generate particular atmospheres, which arise through the confluence of people, objects and experiences in space.

These atmospheres are dynamic, always in a process of emerging and being transformed through lived experiences (Anderson, 2016). Atmospheres are also generative of particular events, actions, feelings and emotions (Bissell, 2010). If the routine patterning of everyday life is significant in constituting the atmosphere and experience of domestic space (Pink and Mackley, 2016), then changes to these rhythms have the potential to remake the atmosphere of the home. It then becomes important to ask what atmospheres are created in everyday domestic space, how different practices shape these atmospheres (Bille and Simonsen, 2019) and how atmospheres may also restrict certain practices (Bissell, 2010).

Thinking beyond the local ensembles through which atmospheres are produced brings into view the wider structures of feeling within the COVID-19 crisis. A structure of feeling is ‘a collective mood that exists in complex relation to other ways in which life is organised and patterned’, a way of thinking and living in a particular time and place that is shared and cuts across different domains of life (Anderson, 2016: 116). Such broader affective conditions within society can be intensified around particular sites and people (Harris et al., 2019); wider collective anxieties and uncertainties of the pandemic can therefore be intensified in certain spaces. This takes affective life beyond the individual subject, setting limits on action by giving sites and encounters a particular ‘feel’, whilst acknowledging the way in which atmospheres envelop and are expressed through particular ensembles of bodies in space (Anderson, 2016).

## Methods

The research aimed to widen conceptual and empirical understandings of the lived experience of a diverse range of small homes in the UK. The overarching research question was: how do individuals experience everyday life in a small home during lockdown, and how does this compare with life before? There is no universally agreed definition of what constitutes a ‘small’ home; in the UK, property size is commonly expressed by number of bedrooms, not floor area (Drury, 2008), and does not account for the feeling of space. Given these contestations, we gave primacy to residents’ perceptions, asking them ‘do you feel like you live in a small home?’, whilst also targeting recruitment at particular house types. For example, a one-bedroom micro apartment may provide less living space per person than a large room in a shared house, but it may feel qualitatively larger because of greater privacy and autonomy. Conversely, a newly built three-bedroom home may not appear ‘small’, but there are longstanding debates around shrinking room sizes and the utility of third bedrooms (Drury, 2008). Research estimates that flats and small terraced houses are most commonly undersized, while households with children are most likely to be overcrowded (Morgan and Cruickshank, 2014). This suggests the need to consider a range of housing and household types. A typology of small homes was developed to underpin a purposive sampling strategy focusing on: new-build micro apartments; new-build ‘family’ homes; older terraces and tenements (Victorian- and Edwardian-era walk-up flats); and house/flat shares.

The cities cover a range of housing markets in which these forms of dwelling were clustered. London and Edinburgh are both capital cities with pressured housing markets, whilst Sheffield has house prices and incomes closer to the national average, with a large stock of older dwellings built to house the working classes during the Industrial Revolution. Due to the COVID-19 restrictions outlined in the introduction, recruitment and interviews were carried out remotely and by telephone (a method used successfully in previous research; Soaita and McKee, 2019). The shared experience of the pandemic, of working from home and juggling caring commitments, helped to generate rapport with participants, despite physical distance. Following ethical approval, two channels were used for recruitment. Internet-based recruitment, via the research organisation’s social media, newsletters and website, invited potential participants to make contact; this was the key route to recruiting individuals living in older homes and flat shares. To locate households in small new-build flats and ‘family’ homes, specific developments were also identified within each city, targeting smaller homes and those near or below (English) space standards. To determine this, planning applications and information on floor space within sale and rental data for newly built properties were consulted and, using the Royal Mail address finder, tailored letters were sent to identified ‘micro apartments’ and smaller ‘family-sized’ newbuilds. For the latter, letters were sent to three-bedroom properties, and households with children were particularly encouraged to contact us. This was to ensure the inclusion of households with children, who could be living in smaller homes but were less likely to be represented among micro apartments and house shares. Participants received a £25 shopping voucher.

When individuals first made contact, data were collected to support the purposive sampling approach, including household composition, a description of the dwelling and perceptions of house size. We were not contacted by anyone who did not feel that they lived in a small home, and therefore no exclusions were made. However, to ensure diversity of sample within the confines of the project budget and scope, once a number of participants had been reached in a particular location and/or house type, a waiting list was put in place while recruitment continued for other cities, households or house types. In line with other research into everyday rhythms (Lager et al., 2016; Rinkart, 2020), semi-structured interviews were conducted with 27 individuals, generating a diverse sample in relation to tenure, house type and household composition (see Table 1).

**Table 1.** Participant characteristics.

Age	
18–24	1
25–34	14
35–44	6
45–54	4
55–64	1
64+	1
Sex	
Male	8
Female	19
Ethnicity	
White British	17
White other	4
Mixed white and black Caribbean	1
Pakistani British	1
Bangladeshi British	1
Other Asian background	3
Area of employment (self-described)	
Policy, research and charity sector	7
Design, marketing and recruitment	4
Education and health	4
Local or national government	3
Project management and consultancy	3
Facilities and building maintenance	2
Student	2
Looking for work	2
Working at home during lockdown	
Yes (completely or partly)	23
No	2
Loss of employment within lockdown period	2
Household composition	
Lives alone	9
Lives with partner/spouse	5
Lives with friends/others	5
Single person with child(ren)	3
Couple with child(ren)	3
Multi-generational household	2
City	
London	13
Sheffield	9
Edinburgh	5
Dwelling type	
House/flat share (friends/strangers)	4
Old flats	3
Old terraces or tenements	5
New-build micro apartments	11
New-build family homes	4
Tenure	
Private rented sector	11
Social rented sector	2
Owner-occupier	14

As may be expected given intergenerational inequalities (Hoolachan and McKee, 2019), a higher proportion of younger people participated. The English Housing Survey suggests that older households have significantly more floor space than younger households, with those aged 65 or over having almost twice as much useable space as 25–34 year olds (Judge and Rahman, 2020). Those aged 25–34 are also almost twice as likely to lack access to a private garden as those over 65 (Judge and Rahman, 2020). The experience of living in a smaller home is therefore differentiated, with impacts falling unevenly on different groups (McKee et al., 2017).

Participants received an electronic copy of a participant information sheet and consent form, which was completed at the start of the call. Interviews were structured by a topic guide, which covered arrival stories, housing choice and aspirations (discussed in a separate publication), everyday life and experiences of lockdown. Interviews particularly sought to draw out the relationship between perceptions of space in the home and the conduct of daily life, and the changes brought about by lockdown to the use and perceptions of the home. Interviews lasted between 45 minutes and 2 hours, and were recorded and fully transcribed. Analysis was carried out in line with constructivist grounded theory approaches (Hoolachan and McKee, 2019), comprising line-by-line coding, theory construction and re-coding in a bottom-up, iterative process. The theoretical framework of urban rhythms was utilised as an analytical tool to provide a wider architecture within which to situate and understand the transformation in daily life arising from the pandemic. A theory foregrounding the relational, temporal and spatial was particularly appropriate to the themes arising from the data because COVID-19 disrupted and restructured routines, relationships and the spatial patterning of everyday activities.

## Findings

### Pre-lockdown: Negotiating polyrhythms and leaving the small home

Participants were asked to reflect on their daily life in ‘normal’ times, before the pandemic, considering the challenges and potential benefits to living in a smaller home. As residents circulated through dwelling space, they engaged in spatial and relational negotiations which compressed or expanded the available space of the home depending on how routines intersected. This was particularly evident in shared homes, with such processes a feature of a range of living environments (Muñoz, 2018). For Isabelle (25, private rented sector (PRS), large house share, London), the kitchen was ‘redundant to me at certain times of the day … If someone’s in the living room, I’m not going to go and use the living room.’ This meant that the space of the home:

sometimes messes up with my routines … If … I’ve come back from a busy day … and I want to go in and cook in the kitchen I can’t because it’s quite small … If someone’s in there I’m just not going to eat until like probably gone 9 o’clock. (Isabelle)

Harry (24, PRS, large house share, London) similarly explained that his use of the kitchen was ‘very dictated on obviously work … One of my housemates … we did work very similar hours … we would be up at the same time, we would be cooking at the same time.’ Even in small flat shares with one other friend, participants worked around the routines of other residents because ‘someone else is in this space and so you always have to kind of plan your time and navigate around that’ (Helen, 31, PRS, small flat share, London).

These kinds of tensions have always existed in diverse shared homes internationally (McKee et al., 2017; Muñoz, 2018), but within small homes it can be seen that the negotiation of polyrhythms gives rise to particular routine practices among family units and couples, not just in flat-share and multi-household arrangements. Even when others were absent, the residue of their use of space was a visible reminder of the pressure on living space, from washing up, to laundry and laptops. Maria (53, social rented sector (SRS), two-bed flat, London) described the ‘challenges’ of a small home: ‘I always feel that I have to move things. I can’t really leave the sewing machine there, all my stuff.’ Similarly, Laura (31, owner-occupier (OO), micro apartment, London) explained that ‘even if you just leave … one bowl on the side … before you know it … the whole thing is a mess’. The home was therefore less able to accommodate different uses, creating a sense of movement as objects were shifted to make way for other functions. As King (2004: 173) argues, dwelling is a tacit relation, something we notice only when it ceases to function as we expect.

Rhythms are not just related to the use of space but are also a multisensory experience, particularly for those living in close proximity to others (Rinkart, 2020). Daily routines that clashed with the dominant pattern could create conflict: ‘I was working nights, and nobody understands that … Normal noises wake you up … Then you would get people … talking to you. They don’t realise that is like morning rush for you’ (Valentina, 49, PRS, studio, London). For some, the friction of polyrhythms extended beyond the dwelling, as participants came into contact with contrasting rhythms governing daily life:

In a flat you’re … in a massive block with loads of other people … you’re hyper-aware to … what’s going on around you, whether it be neighbours making loud noises or … people throwing rubbish … You’re so close to other people around you that you can never fully relax. (Heather, 26, SRS, two-bed flat, London)

Close proximity to others demonstrates how the practices of one neighbour can impede the home-making of another (Cheshire et al., 2021), as Heather struggles to relax due to the clash of different routines, which creates a multisensory atmosphere of heightened vigilance.

By trading off space in the home, living somewhere small enabled access to a particular urban lifestyle and set of amenities. However, limited living space also encouraged residents to spend more time outside the home, reinforcing their engagement with the rhythms of urban living, regardless of its desirability, and this was evident across different tenures and household types. As Sophie (41, OO, tenement, Edinburgh) explained when talking about life in a small home, ‘I don’t sit in my flat, I’m never here.’ For many, space was not seen as an issue because life unfolded outside the home: ‘our social life is … out and about and it’s doing things … going to see things … It’s a place to sleep’ (Tom, 29, OO, micro apartment, London). Whilst it was a key part of participants’ housing choices ‘to be able to do things and kind of live in a more exciting city’ (George, 27, PRS, micro apartment, Edinburgh), space in the home could also influence patterns of sociability. Isabelle (25, PRS, large house share, London) explained negotiating the routines of other residents: ‘Because people are kind of occupying the living room or the kitchen … I can’t use those spaces, so I’d much rather go out for dinner.’ Similarly, Harry (24, PRS, large house share, London) noted that ‘you’re kind of forced to have to go out to do it [socialising] … you would go down the pub rather than just chill in your house’.

Daily rhythms were therefore influenced by the experience of home and the wider urban environment, as individuals engaged with the ‘eventfulness’ of the city (Kern, 2015). Leaving the home was a key way of managing life in a small home. As Bilal explained, he adjusted his routines to avoid other residents and spend little time in his room:

I don’t want to interact as much with people … that’s why I say very little, I do very little in the property … Before six o’clock I use the kitchen to cook a meal … Then go back in the room … go to work … come back home at five or six and that’s it. (Bilal, 30, PRS, house in multiple occupation (HMO), Sheffield)

The rhythm of his day was therefore based on routinised practices of distancing from other residents and ensuring access to the shared spaces that were crucial for meeting basic needs, much like residents of informal housing (Muñoz, 2018).

### Lockdown and arrhythmia in daily life

Participants were asked to consider whether the way in which they used their living space had changed as a result of the COVID-19 lockdown. Whilst inequalities in space could be masked in ‘normal’ times by engaging with wider neighbourhood life beyond the home, the requirement for most of the UK population to remain at home disrupted habitual patterns of daily life. Lockdown intensified existing pressures faced by those at the sharp end of inequalities in living space, at the same time as restricting adaptive mechanisms – such as being outside the home – that provided respite. Given that individuals were used to much more fluidity between private domestic space and their wider neighbourhoods, the requirement to stay at home forced a sudden shift in the patterning of daily life, and the residue of ‘normal’ rhythms (Buttimer, 1976) was apparent in discussions.

The home had to cater to more functions and the prolonged presence of household members. Only two participants – both doctors – were not working from home at all during the lockdown. Shared spaces were more difficult to negotiate with expanded households; for example, when Olivia’s boyfriend moved in before lockdown, the main place ‘we sort of notice it is … in the kitchen or in the lounge … because the space just is quite … tight’ (Olivia, 23, PRS, new-build house, Sheffield). Others found that new rhythms of daily life were suddenly changed:

My boyfriend had only moved in at the start of March so we’d lived together for … three weeks? And then lockdown was imposed … We’d kind of begun to establish a pattern … and then suddenly … you’re trapped in this box, all the time. (Sarah, 25, PRS, micro apartment, Edinburgh)

For some, finding private spaces became more important; one participant reflected that ‘I can retreat into this very small bedroom … We need more of our own space than what we did before, because we’re spending so much time together’ (Amy, 46, PRS, small older terrace, Edinburgh).

Tacit ways of knowing and navigating the home (Pink and Mackley, 2016), which provide a sense of familiarity, were exposed, necessitating new negotiations between household members as they circulated around the home:

We’re [my mother and I] both at home … I work from my bedroom. She likes to go out for walks … Not really a schedule, but we’re able to like do our own thing and not get on each other’s toes … We’re able to … have that distinction between like I’m actually … doing my work and I’ve got to concentrate. And she’ll go and do her own thing … maintain that distinction and space. (Heather, 26, SRS, two-bed flat, London)

A small space required more work to differentiate spaces and meet the needs of different household members. Helen (31, PRS, small flat share, London) explained that working at home with her flatmate required them to ‘learn the ways of working from home and get into the rhythm of that’.

The sudden change to daily routines was particularly acute for those in small homes because they lacked spatial flexibility. Maria (53, SRS, two-bed flat, London) explained that ‘before the pandemic this dynamic was okay because my daughter went to school, I went to work part time, my other daughter as well, my son was working … Everything has changed.’ The lockdown exposed the fragility of the coping mechanisms that enabled life to function. The impact of caring responsibilities added to tensions around sharing space and resources, as people and objects circulated through the home: ‘We just do different things at different times. We organise ourselves … I say, “I don’t need a laptop now”, so my daughter [can] have it. And then she has a break and I’ll have it’ (Maria). With schools closed, families tried to adapt spaces to new functions: ‘home learning will always happen in … our kitchen-dining-lounge … our family space. But also … I can retreat into this very small bedroom’ (Amy, 46, PRS, small older terrace, Edinburgh). Separate spaces provided crucial respite from the stresses of managing work, home and caring roles. Julie (43, OO, small older terrace, Edinburgh), who had a garden, noted that it had allowed the family to manage stresses: ‘if there’d … been home schooling … getting a bit irritating … I’ve just gone off and done something in the garden’.

This circulation and movement generated work in which:

we shift a lot … My daughter goes to … the bedroom, and then has a break, goes to the sitting room … Then she comes in here [Maria’s bedroom] … So I move out. It’s a lot of moving around … Rotating, going up and down … A lot of moving and shifting. (Maria, 53, SRS, two-bed flat, London)

Adapting to different functions therefore added to the work of living in a small home. This was similarly expressed by other participants in relation to working at home and video conferencing, which required the negotiation of household members and the shifting of objects. Thus, Jackie described ‘needing to actually physically move everything into the bedroom … so we’re not disturbing each other’ (Jackie, 63, OO, tenement, Edinburgh), whilst Eleanor explained that:

you have your breakfast … you move the bowl away and then you have to put your laptop there to do work. And then you move your laptop because you want to have a sandwich on a plate … and then you move that. (Eleanor, 35, OO, micro apartment, London)

### Exception and home atmospheres

The lockdown acted as a mechanism of exception (Stavrides, 2013), imposing new rhythms; in reflecting on whether their feelings about their home had changed during lockdown, participants noted the way in which new routines – particularly related to home working – had affected their home life. This was not just about lack of physical workspace but also about emotional separation, as those in small homes were less able to create spaces for different rhythms, creating an atmosphere of monotony: ‘It goes in waves … I think it really gets to me especially … during the weekends … you just want to do something different … Everything’s all merged into one … I literally wake up, have breakfast, switch on my laptop and I’m at work’ (Heather, 26, SRS, two-bed flat, London).

Atmospheres are related to ‘forms of enclosure’– such as rooms – and circulation, as they surround and radiate through space (Anderson, 2009). Those living in smaller homes during a state of exception, typified by restricted movement, experienced particular ensembles of people and objects in limited space, impacting on home atmospheres: ‘Myself and my boyfriend … are the only ones [working] … everyone else is kind of just chilling around the house … so you’ve got to go to your room’ (Isabelle, 25, PRS, large house share, London). Atmospheres can facilitate or disrupt particular practices; just as Bissell (2010) analyses the way in which the train carriage can take on an atmosphere of work during daily commuting hours, so here the chilled atmosphere of shared spaces precluded a work atmosphere. Atmospheres can therefore ‘leak out’, permeating space and radiating between individuals (Anderson, 2016).

Tom (29, OO, micro apartment, London) explained that his wife had been furloughed from her arts sector job. He was conscious that the increasing dominance of his work rhythms was a reminder of his wife’s change in work status:

The more established that that workspace has got and the more suitable it’s got, the more it dominates … I don’t want … our shared living space to become my workspace … I don’t want my poor wife sort of sat in the bedroom waiting to come out … It’s a really awkward thing to manage.

This kind of ‘unspoken encounter’ is part of producing the affective atmosphere of the home (Pink and Mackley, 2016), through which the production of work rhythms acts to exclude other household members and functions. Agata (26, PRS, micro apartment, Edinburgh) described similar challenges:

My partner is mostly occupying the living room … He gets to have access to the kitchen … and I’m just working in a bedroom all day … Sometimes there are difficult moments, like … I need a living room space … and I … have to … ask if I could.

The atmosphere of the home therefore communicates the sense of belonging or not (Anderson, 2016), as shared spaces become redefined through working practices.

The normal rhythms of the daily commute and sociability which differentiated time and space before COVID-19 were lost, and the merging of home and work practices impacted on the sense of ‘feeling right’ at home (Pink, 2012). As Isabelle explained, ‘I didn’t really want to be working just in my bedroom because … it’s my relaxing space; I do want to have that kind of separation’ (Isabelle, 25, PRS, large house share, London). Similarly, Beth (36, OO, micro apartment, London) found that her laptop on the table was ‘a permanent reminder’ of work, highlighting the way in which people and objects interact in particular spaces to generate affective atmospheres (Anderson, 2009). Some tried to create separation by moving items, as Jo (34, OO, new-build house, Sheffield) explained: ‘we’ve had to sacrifice one of the shelves and get rid of some of the stuff … and that’s become kind of my workstation area’.

The sense that ‘everything’s sort of merged into one’ (Heather, 26, SRS, two-bed flat, London) also impacted on wellbeing. Sarah (25, PRS, micro apartment, Edinburgh) was ‘having real problems sleeping … because I had like a routine that I was used to … I did have to … try to get back into a routine … find a way to separate weekdays from weekends’. The inflexibility of living in a smaller space could change the feel of the home because things were ‘a little bit more oppressive if you’ve got the same few walls and the same limited space’ (Tom, 29, OO, micro apartment, London). Living in ‘quite a confined space … you kind of crave to go out’ (Hannah, 30, OO, micro apartment, London). The disruption to usual routines and coping strategies was difficult for those in the smallest shared homes: ‘I always used to be outside before, I was at work … I used to go out during the day … It … plays with you emotionally’ (Bilal, 30, PRS, HMO, Sheffield).

At the same time as wellbeing was strained, the rules of lockdown disrupted the strategies that individuals used to manage their mental health. Robert noted that ‘you’re not aware of how it does get to you if you are on your own … When I get up in the morning … there is only me to say hello to’ (Robert, 69, OO, micro apartment, Edinburgh). Similarly, Sophie usually spent most of the time outside her home:

I never spent a single full day in my house, I’ve never spent a weekend here … I don’t want to start off … problems with depression and feeling isolated, and all of the stuff that I try and put in place, fitness … doing the bar job … All that apparatus you just see it go out the window. (Sophie, 41, OO, tenement, Edinburgh)

Some attempted to regain some of the lost rhythms of their ‘normal’ life, for example through meeting others. Robert explained that ‘I did break the rules … going for walks with [my son] and one or two other people … I needed to see some human beings … For me personally, there is a balance of sanity against the infection’ (Robert, 69, OO, micro apartment, Edinburgh). Similarly, Valentina (49, PRS, studio, London) explained that:

just meeting one friend, it would save your mental health … I am here on my own and my friend is in the same situation, we have decided we are going to be meeting … We don’t think we are risking anybody else’s life.

This highlights the presence of ‘counter-rhythms’ (Reid-Musson, 2018) even in a time of exception; in deploying these ‘tactics’ (Stavrides, 2013), individuals sought to circumvent the new dominant rhythms.

Wider structures of feeling can be intensified in particular sites (Harris et al., 2019), and whilst participants faced monotony, anxiety and uncertainty that were shared across different domains during the pandemic, the way in which these were lived within small homes was perceived as different from the way they were lived for those with more space. A number of participants reflected on the way in which inequities in space created a differentiated experience of lockdown. Maria (53, SRS, two-bed flat, London) explained that she found it ‘really challenging when other people used to say “oh it feels like this and that”, and yet they had far more space. They had their own office in their home and a big garden.’ Those without outside space described observing those who were able to change the patterning of their day by moving between outside and inside space: ‘the view of my window is directly into blocks of flats and they’ve got … the shared garden … it’s causing some garden envy’ (Isabelle, 25, PRS, large house share, London). Similarly, Heather (26, SRS, two-bed flat, London) noted that ‘there’s lots of focus on the news with “oh, you can have people around if you’ve got a garden” … But what they fail to realise is that lots of people don’t have the luxury of having that.’ Outside space was particularly problematic for those with no private garden, an issue that was magnified in cities at a time when access to public spaces was restricted.

## Discussion and conclusion

Situating the spatiality of small-home living within a framework of urban rhythms and atmospheres has demonstrated the way in which home space patterned the lives unfolding within and beyond the dwelling. Everyday life in a small home already involved compromise and negotiation, but the COVID-19 pandemic suggests the utility of thinking of small homes as sites of intensification in which existing challenges were exacerbated. The research aligns with the notion that living conditions had greater impacts on wellbeing than before the pandemic (Judge and Rahman, 2020), because adaptive strategies were disrupted. Wider structures of feeling in the pandemic were intensified in particular sites (Harris et al., 2019), with participants in small homes reporting a sense of life merging into one, boredom and in some cases anxiety and stress, exacerbated by the inability to vary their use of space. The disruption and confinement of lockdown helped to reveal the compromises associated with living in a smaller home, many of which occur in relation to mundane daily practices (Muñoz, 2018).

In ‘normal’ times, routines reflected the value that residents attached to living in particular neighbourhoods, which offered access to amenities and cultural life; everyday rhythms were governed by movement to and from work, social spaces and home (Kern, 2015; Reid-Musson, 2018). Daily life was therefore characterised by a particular spatial form, with different activities performed in settings within and beyond the home, including in public space, schools and hospitality venues (Aquist, 2004). Given the importance of routines outside the home, it is not surprising that some of the most negative impacts of lockdown would be experienced disproportionately by those in more marginal housing, including small spaces (Brown et al., 2020). Lockdown unpicked adaptive strategies, as homes became a place of work, school and wider sociability, accommodating more and varied functions. With limited space, there is less flexibility to redefine parts of the home to meet household needs.

In conceptualising lockdown as a ‘mechanism of exception’ (Stavrides, 2013), disrupting routines of life in service of a wider public objective, this research situates the aberration of COVID-19 within a longer history of other moments of disruption. The concept leaves open the possibility of a more fundamental reshaping of rhythms of daily life, as over time the imprinting of a new rhythm can reshape normative rules about behaviours, practices and identities (Kern, 2015: 444). For example, during lockdown the new regulation of mobility (Cresswell, 2010) meant that many aspects of contemporary urban life lost their value as new norms of work and social life were embedded.

Whilst the longevity of these changes remains to be seen, in linking the concepts of exception, rhythms and atmospheres, the research shows how the enforced disruption to habitual routines changed home atmospheres by transforming the relationship between people, objects and practices in home spaces. New atmospheres may be apprehended visually (Bissell, 2010) or through other modes such as the presence or absence of noise or objects (like work equipment), being produced across human and non-human materialities (Anderson, 2016). Lack of space created the conditions for conflicting atmospheres, boredom and isolation, as individuals were unable to change the rhythm of the day by moving between places characterised by a different feel. Although individuals may adjust to this ‘new normal’, there were also instances of counter-rhythms (Kern, 2015) in which participants inserted ‘hidden rhythmicalities of survival based on disguised and protected habits’ (Stavrides, 2013: 42), such as when Robert continues to meet his son and others for walks as a way of protecting his own mental wellbeing. This is likely to increase in significance as people and places move in and out of more and less stringent restrictions, and compliance wanes.

Buttimer (1976) argues that the ‘residue’ of former rhythms influences the evaluation of new environments, resonating with the ways in which individuals talked about disruption to their usual routines, and perception that others were continuing to engage in mundane activities that were not open to all. Whilst observing the unfolding of everyday life may enable individuals to experience the vitality of the neighbourhood, bridging the gap between different rhythms (Lager et al., 2016), during lockdown such observations further highlighted participants’ marginalisation and the sense of discontinuity with life before. For many, the pandemic has magnified the sense that key markers of ‘normalcy’– expressed through housing – have not been achieved. For some, policies designed to ease the burden of lockdown – such as meeting in private gardens (Blackall, 2020) – only highlighted inequalities in domestic space. However, these conversations also indicated that there were commonalities of experience across different households, in different housing market positions and life stages, united by the everyday reality of living in a smaller space. Attention at the level of rhythms therefore has potential to reveal intersecting positions, as well as differential experiences, which may otherwise be hidden (Reid-Musson, 2018).

Drawing on the framework of urban rhythms has highlighted the exceptional disruption to the normal patterning of daily life, impacting on practices within the home as well as the affective qualities that are produced within it. Future research incorporating more dynamic methods would be of value, particularly capturing change over time, for example through the use of timelines or diaries. This could generate a more in-depth understanding of the rhythms of daily life, as well as enabling consideration of the extent to which ‘exception’ becomes normalised through the entrenchment of new routines over time. Given the negotiation that is involved in living in a small home – not just within flat-share arrangements, but also within family units and couples – there is also potential for methods which take a whole household approach in order to draw out the perceptions of household members.

The research indicates that for those living in smaller homes the relationship with the wider neighbourhood and its amenities is important, providing balance to smaller living spaces. Further research should focus on expanding understandings of the relationship between the positive ‘pull’ factors that facilitate engagement with wider neighbourhood life, as well as the negative ‘push’ factors that may prompt individuals to leave the home. Finally, consideration may also be given to larger scale comparative work among different households and types of small home. This would develop research findings presented here, which indicate that there are commonalities of experience among those living in smaller homes, despite different social and housing market positions.

Whilst conceptualising the COVID-19 pandemic as a mechanism of exception, the immediacy of the lockdown accelerated and exacerbated the impact of a more gradual reduction in domestic living spaces over time. Yet, the strategies used by residents to manage small-home living in ‘normal’ times are not permanent fixtures equally available to all, but are vulnerable to changes in urban society, the built environment and personal circumstances. For example, a prolonged move to homeworking may weaken the desirability of smaller homes for some. As such, the findings presented here have relevance beyond the specific context of the pandemic and, linked to the future research agendas highlighted above, suggest the potential for renewed attention to the dynamic adaptations – both practical and symbolic – that individuals make to constrained living spaces.
